# Effect of Prestroke Glycemic Variability Estimated Glycated Albumin on Stroke Severity and Infarct Volume in Diabetic Patients Presenting With Acute Ischemic Stroke

**DOI:** 10.3389/fendo.2020.00230

**Published:** 2020-04-21

**Authors:** Sang-Hwa Lee, Min Uk Jang, Yerim Kim, So Young Park, Chulho Kim, Yeo Jin Kim, Jong-Hee Sohn

**Affiliations:** ^1^Department of Neurology, Chuncheon Sacred Heart Hospital, Hallym University College of Medicine, Chuncheon, South Korea; ^2^Department of Neurology, Dongtan Sacred Heart Hospital, Hallym University College of Medicine, Hwaseong, South Korea; ^3^Department of Neurology, Kangdong Sacred Heart Hospital, Hallym University College of Medicine, Seoul, South Korea; ^4^Department of Endocrinology and Metabolism, Kyung Hee University Hospital, Seoul, South Korea

**Keywords:** glycated albumin, glycated hemoglobin, acute ischemic stroke, diabetes mellitus, severity, infarct volume

## Abstract

**Background:** We investigated whether prestroke glycemic variability, represented by glycated albumin (GA), affects the initial stroke severity and infarct volume in diabetic patients presenting with acute ischemic stroke.

**Methods:** We evaluated a total of 296 acute ischemic stroke patients with diabetes mellitus who were hospitalized within 48 h of stroke onset. GA was measured in all acute ischemic stroke patients consecutively during the study period. The primary outcome was the initial National Institute Health Stroke Scale (NIHSS) score. The secondary outcome was infarct volume on diffusion-weighted imaging, which was performed within 24 h of stroke onset. Higher GA (≥16.0%) was determined to reflect glycemic fluctuation prior to ischemic stroke.

**Results:** The number of patients with higher GA was 217 (73.3%). The prevalence of a severe initial NIHSS score (>14) was higher in patients with higher GA than in those with lower GA (3.8% vs. 15.7%, *p* = 0.01). The proportion of participants in the highest quartile of infarct volume was higher in the higher GA group (11.4% vs. 36.4%, *p* < 0.001). A multivariable analysis showed that higher GA was significantly associated with a severe NIHSS score (odds ratio, [95% confidence interval], 7.99 [1.75–36.45]) and large infarct volume (3.76 [1.05–13.45]).

**Conclusions:** Prestroke glucose variability estimated by GA was associated with an increased risk of severe initial stroke severity and large infarct volume in acute ischemic stroke patients with diabetes mellitus.

## Introduction

Various glucose parameters have been used to predict poor stroke outcomes but remain controversial (1). Several studies have shown that glycated hemoglobin (HbA1c), reflecting prestroke chronic hyperglycemia, was associated with poor functional outcomes after stroke (2–4). Nonetheless, a recent experimental study showed that glycemic variability could trigger more oxidative stress and both microvascular and macrovascular injury than chronic hyperglycemia (5, 6). Hence, prestroke glycemic variability could be more associated with stroke outcomes than prestroke chronic hyperglycemia.

A consensus on the gold standard method to measure glycemic variability in clinical practice and research is still lacking (1). The easiest way to measure glycemic variability is to estimate the standard deviation (SD) of the mean blood glucose and the coefficient of variation (CV). However, since this method requires data from at least 2 consecutive days after stroke onset to perform the estimation, SD and CV could not be appropriate indexes of prestroke glycemic variability to predict outcomes of acute ischemic stroke.

Glycated albumin (GA) reflects glycemic variability within 4 weeks of stroke onset (7, 8), so GA could be a useful marker for predicting prestroke glycemic variability. Additionally, GA is measured quickly and easily with one blood sample.

The relationship between prestroke glycemic variability and initial stroke severity and infarct size on diffusion-weighted imaging (DWI) has not been assessed previously. In this study, we investigated the effects of glycemic variability measured by GA on stroke severity and infarct volume on DWI compared with using HbA1c in individuals with diabetes mellitus (DM) presenting with acute ischemic stroke.

## Methods

### Subjects

We prospectively registered all acute ischemic stroke patients between March 2016 and May 2019 in our institution. For the purpose of this study, we included only patients who had an established diagnosis of DM (HbA1c value ≥ 6.5%) (9) at the time of hospitalization or who had a history of or used hypoglycemic agents or insulin. In this study, we excluded the following patients: (1) patients with unavailable GA and brain magnetic resonance imaging (MRI) data. (2) Patients with transient ischemic attacks without ischemic lesions visible on MRI performed within 24 h of stroke onset.

### Data Collection and Definition of Parameters

The following data were directly obtained from the registry database: (1) Demographics including age and sex; (2) stroke risk factors, medical history, prior stroke, hypertension, DM, hyperlipidemia, atrial fibrillation, current smoking, and prior use of antithrombotic drugs; (3) stroke characteristics, acute stroke treatment, initial NIHSS score, ischemic stroke mechanism according to the Trial of Org 10172 in Acute Stroke Treatment (TOAST) classification, and reperfusion therapy; (4) laboratory data including hemoglobin, creatinine, initial random glucose, total cholesterol, low-density lipoprotein, high-sensitivity C-reactive protein (hs-CRP), HbA1c, serum albumin, and systolic blood pressure.

The primary outcome measure was the categorized initial stroke severity assessed by NIHSS score at the time of hospitalization (mild: NIHSS 0–5, moderate: NIHSS 6–14, and severe: NIHSS > 14) (10, 11). The secondary outcome measure was the ischemic lesion volumes on DWI calculated by Medical Image Processing and Visualization software (MIPAV, version 7.3.0, National Institutes of Health, Bethesda, MD). Acute ischemic lesions were identified on a slice-by-slice basis and were correlated with low signals on apparent diffusion coefficients. DWI infarct volumes were calculated by multiplying slice thickness by total areas of lesions. Two experienced neurologists (S-H Lee and MU Jang) assessed all infarct volumes on DWI (interclass correlation coefficient = 0.87). The infarct volumes were divided into quartiles and denoted as Q-25, Q-50, Q-75, and Q-100. Q-25 indicated the lowest quartile of DWI infarct volume, and Q-100 indicated the highest quartile.

### Measurement of GA Level

Venous blood was drawn from fasting patients within 12 h after admission. The serum specimens were collected and measured by an enzymatic method using albumin-specific proteinase and ketoamine oxidase (Lucica GA-L; ASAHI KASEI PHARMA, Japan) (12). Several studies suggested the cutoff point of GA to identify DM (8). A study with a large population (*n* = 2,192) in Taiwan described a cutoff point of GA ≥ 14.5% for DM. In addition, when the value of 6.5% of HbA1c was considered, the corresponding GA was 16.5%. Another study with a relatively large population (*n* = 1575) in Japan described the cutoff point of GA ≥ 15.5%. Hence, we established that a GA level ≥ 16.0% reflected the presence of glycemic variability prior to ischemic stroke based on the following equation of HbA1c and GA: HbA1c = 0.216 × GA +2.978 (13). According to GA levels, we divided the population into a lower GA group (GA < 16.0%) and a higher GA group (GA ≥ 16.0%).

### Statistical Analysis

We assumed that higher GA levels, which indicate high glycemic variability prior to the stroke, could be associated with initial stroke severity and infarct volume.

Summary statistics are presented as the number of subjects (percentage) for categorical variables and as the mean ± SD or median (interquartile range, IQR) for continuous variables. Group comparisons were made using Pearson's chi-squared test for categorical variables and Student's *t*-test or the Mann-Whitney U-test for continuous variables, where appropriate.

With respect to the primary and secondary outcome measures, the lower GA group and the higher GA group were compared using Pearson's chi-squared test, and independent effects of GA on those outcome measures were analyzed using multinomial logistic regression and multiple linear regression models. Variables for adjustment in the multivariable analysis were selected if their *p*-values were <0.2 in comparisons according to the GA level and if their associations with each outcome variable were clinically plausible. Crude and adjusted odds ratios (ORs) and 95% confidence intervals (CIs) were estimated.

In a sensitivity analysis, we also analyzed whether HbA1c could affect primary and secondary outcomes. Crude and multivariable analyses were performed according to dichotomized HbA1c (<6.5% vs. ≥ 6.5%). In addition, we included the ratio of GA to HbA1c (GA/HbA1c ratio) in multinomial logistic regression analysis because the GA/HbA1c ratio could reflect more accurate glycemic control (14). The impact of elevated HbA1c and GA/HbA1c ratio on outcomes was assessed by multinomial logistic regression analysis. Moreover, we categorized GA and HbA1c into tertiles and analyzed the effects on outcomes.

In a subgroup analysis, we analyzed whether GA could affect outcomes according to stroke mechanisms (large artery atherosclerosis [LAA], small vessel occlusion [SVO], and cardioembolism [CE]). Since the sample size was small for each stroke mechanism, we dichotomized NIHSS scores as mild (NIHSS 0–5) and moderate to severe (NIHSS 6–42) (11). All data analyses were performed with IBM SPSS version 21.0 software (IBM Corporation, Armonk, NY, USA).

## Results

Among the 761 consecutive patients with acute ischemic stroke, 296 patients diagnosed with DM (175 males and 121 females, aged 70.4 ± 11.5 years) were included in the study throughout the study period. Of the 296 patients, 217 (73.3%) were in the higher GA group (GA ≥ 16.0%) and 203 (68.6%) were in the high HbA1c group (HbA1c ≥ 6.5%). The higher GA group was more likely to have a higher HbA1c level, initial random glucose, and larger infarct volume. The higher GA group tended to have more severe stroke symptoms than the lower GA group (*p* = 0.052). In non-diabetic patients (*n* = 465) in the present study, the initial NIHSS score was 3 (IQR 1–6) in the lower GA group and 4 (IQR 1–10) in the higher GA group (*p* = 0.01, data were not shown). We carefully suggest that the higher GA group seems to have more severe stroke symptoms in both DM and non-diabetic patients presenting with acute ischemic stroke. Generally, the stroke characteristics and demographics were not different between the lower GA and higher GA groups (Table 1). Compared with the results according to GA levels, stroke severity and infarct volumes were not significantly different according to HbA1c levels (Supplemental Table 1). Despite well-controlled DM patients having HbA1c levels <6.5%, half had higher GA levels (48.4%). Pearson's correlation analysis showed that the raw GA was significantly positively correlated with both initial NIHSS scores (*r* = 0.20; *p* < 0.001) and DWI infarct volumes (*r* = 0.35, *p* < 0.001) (Supplemental Figure 1).

**Table 1:** Baseline characteristics of the study population (*n* = 296) according to GA levels.

	**Lower GA group (GA <16.0%) *n* = 79**	**Higher GA group (GA ≥ 16.0%) *n* = 217**	***p*-value**
Age, years (SD)	70.8 (11.8)	70.2 (11.4)	0.67^†^
Male (%)	44 (55.7)	131 (60.4)	0.51^*^
BMI, kg/m^2^ (SD)	24.2 (3.4)	24.5 (3.7)	0.82^†^
Interval from onset to visit, hours [median (IQR)]	14.8 (5.3–50.0)	12.9 (3.7–38.4)	0.35^‡^
History of stroke (%)	23 (29.1)	59 (27.2)	0.77^*^
Hypertension (%)	64 (81.0)	158 (72.8)	0.17^*^
Duration of DM, year (SD)	7.3 (2.7)	5.2 (2.7)	0.18^†^
Hyperlipidemia (%)	17 (21.5)	34 (15.7)	0.30^*^
Current smoking (%)	12 (15.2)	28 (12.9)	0.70^*^
Atrial fibrillation (%)	15 (19.0)	39 (18.0)	0.87^*^
Coronary artery disease (%)	6 (7.6)	20 (9.2)	0.82^*^
Prior antithrombotic agents (%)	41 (51.9)	96 (44.2)	0.29^*^
Initial NIHSS score (IQR)	4 (2–6)	5 (2–11)	0.052^‡^
Stroke mechanism (%)			0.14^*^
SVO	27 (34.2)	51 (23.5)	
LAA	28 (35.4)	97 (44.7)	
CE	16 (20.3)	35 (16.1)	
Others	8 (10.1)	34 (15.7)	
Reperfusion therapy (%)	9 (11.4)	28 (12.9)	0.84^*^
Ischemic lesions (%)			0.92^*^
Supratentorial	53 (67.1)	147 (67.7)	
Infratentorial	26 (32.9)	70 (32.3)	
Laboratory data			
Total cholesterol, mg/dL (SD)	153.8 (46.9)	155.2 (43.0)	0.51^†^
Hemoglobin, g/dL (SD)	13.1 (1.9)	13.5 (2.2)	0.48^†^
Creatinine, mg/dL (SD)	1.1 (0.9)	1.1 (0.6)	0.33^†^
Platelets, ×1,000/μL (SD)	228.3 (70.2)	228.1 (80.3)	0.51^†^
hs-CRP, mg/L (SD)	10.8 (27.4)	12.3 (28.8)	0.66^†^
LDL, mg/dL (SD)	51.3 (31.4)	45.9 (31.6)	0.38^†^
HbA1c, % (SD)	6.3 (1.0)	7.6 (1.5)	0.002^†^
Initial random glucose, mg/dL (SD)	150.6 (55.9)	182.7 (74.1)	0.02^†^
Serum albumin, mg/dL (SD)	3.88 (0.30)	3.85 (0.42)	0.08^†^
SBP, mmHg (SD)	150.0 (26.4)	148.6 (25.2)	0.43^†^
Infarct volume, cm^3^, median (IQR)	0.46 (0.21–2.21)	1.77 (0.41–33.15)	<0.001

*GA, glycated albumin; SD, standard deviation; BMI, body mass index; IQR, interquartile range; DM, diabetes mellitus; NIHSS, National Institute Health of Stroke Scale; SVO, small vessel occlusion; LAA, large artery atherosclerosis; CE, cardioembolism; hs-CRP, high-sensitivity C-reactive protein; LDL, low-density lipoprotein; HbA1c, glycated hemoglobin; SBP, systolic blood pressure*.

* *Calculated using the chi-square test*.

† *Calculated using Student's t-test*.

‡ *Calculated using Mann-Whitney U test*.

**Figure 1:**
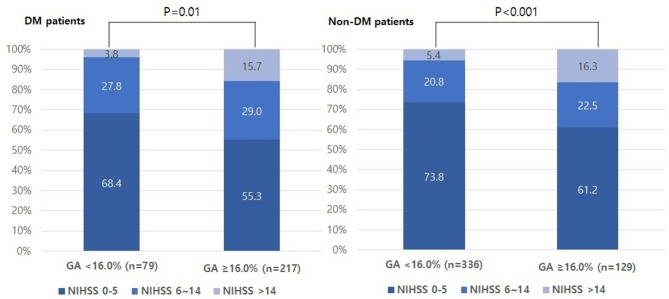
Distributions of initial stroke severity by categorized NIHSS scores according to GA levels in both DM and non-DM patients. NIHSS, National Institutes of Health Stroke Scale; GA, glycated albumin; DM, diabetic mellitus.

**Figure 2:**
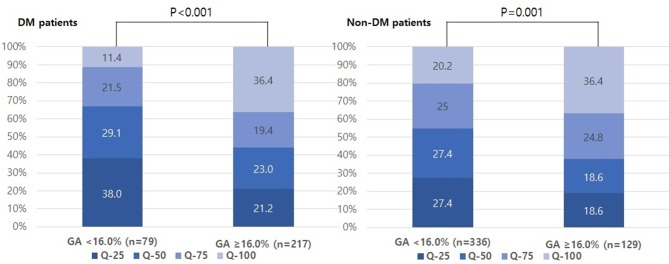
Distributions of quartiles of infarct volumes according to GA levels in both DM and non-DM patients. GA, glycated albumin; DM, diabetic mellitus.

The proportion of individuals with moderate and severe stroke (NIHSS 6–14 and NIHSS > 14) was higher in the higher GA group than in the lower GA group (moderate: 27.8% vs. 29.0% and severe: 3.8% vs. 15.7%, *p* = 0.01, Figure 1). The proportion of individuals in the highest quartile of DWI infarct volume (Q-100) was higher in the GA group than in the GA group (11.4% vs. 36.4%, *p* < 0.001, Figure 2). Additionally, in non-DM patients, the higher GA group had either more severe stroke (NIHSS 6–14 and NIHSS > 14) or DWI infarct volume (Q-100) (Figures 1, 2).

The multinomial logistic regression analysis showed that higher GA levels were significantly associated with severe stroke (adjusted OR, [95% CI], 7.99 [1.75–36.45], Table 2 and Supplemental Table 2). The linear regression analysis showed that raw GA was also significantly positively correlated with initial NIHSS scores (*r*^2^ = 0.16, *p* < 0.001, Supplemental Table 3). Additionally, higher GA was significantly associated with the highest quartile (Q-100) of DWI infarct volumes (adjusted OR [95% CI]; 3.76 [1.05–13.45], Table 3 and Supplemental Table 4). The linear regression analysis showed that raw GA was also significantly positively correlated with initial NIHSS scores (*r*^2^ = 0.25, *p* = 0.002, Supplemental Table 5).

**Table 2:** Multinomial logistic regression analysis: impact of higher GA (≥16.0%) on initial stroke severity (reference = NIHSS 0–5).

	**GA** **≥** **16.0% (*****n*** **=** **217)**		**HbA1c** **≥** **6.5% (*****n*** **=** **203)**		**GA/HbA1c ratio**	
	**Adjusted OR^a^**	**95% CI**	**Adjusted OR^b^**	**95% CI**	**Adjusted OR^a^**	**95% CI**
**NIHSS 0–5**	**Reference**					
NIHSS 6–14	1.25	0.62–2.51	0.88	0.44–1.76	2.27	1.22–4.20
NIHSS >14	7.99	1.75–36.45	2.32	0.76–7.14	5.21	2.11–12.86

*GA, glycated albumin; NIHSS, National Institutes Health of Stroke Scale; HbA1c, glycated hemoglobin; OR, odds ratio; CI, confidence interval*.

a *Adjusted for age, sex, hypertension, stroke mechanism, stroke severity, medications for hypertension, medications for DM, prior antithrombotic agents use, statin use, initial random glucose, serum albumin, and glycated hemoglobin*.

b *Adjusted for age, sex, prior stroke, stroke mechanism, stroke severity, interval from onset to visit, current smoking, prior antithrombotic agents use, atrial fibrillation, reperfusion therapy, medications for DM, previous antithrombotic agents, statin use, creatinine, low-density lipoprotein, glycated hemoglobin and initial random glucose*.

**Table 3:** Multinomial logistic regression analysis: impact of higher GA (≥16.0%) on each quartile of infarct volume (reference = Q-25).

	**GA ≥ 16.0% (*****n*** **= 217)**		**HbA1c ≥ 6.5% (*****n*** **= 203)**		**GA/HbA1c ratio**	
	**Adjusted OR^a^**	**95% CI**	**Adjusted OR^b^**	**95% CI**	**Adjusted OR^a^**	**95% CI**
**Q-25**	**Reference**					
Q-50	1.58	0.65–3.88	2.59	1.04–6.48	0.91	0.41–2.01
Q-75	1.48	0.56–3.97	2.77	1.01–7.62	0.66	0.27–1.64
Q-100	3.76	1.05–13.45	3.39	1.08–10.69	2.95	1.29–6.75

*GA, glycated albumin; HbA1c, glycated hemoglobin; OR, odds ratio; CI, confidence interval*.

a *Adjusted for age, sex, hypertension, stroke mechanism, stroke severity, medications for hypertension, medications for DM, prior antithrombotic agents use, statin use, initial random glucose, serum albumin, and glycated hemoglobin*.

b *Adjusted for age, sex, prior stroke, stroke mechanism, stroke severity, interval from onset to visit, current smoking, prior antithrombotic agents use, atrial fibrillation, reperfusion therapy, medications for DM, previous antithrombotic agents, statin use, creatinine, low-density lipoprotein, glycated hemoglobin and initial random glucose*.

A sensitivity analysis was performed to evaluate the impact of HbA1c on stroke severity and DWI infarct volume. HbA1c ≥ 6.5% was not associated with initial stroke severity but was associated with increasing DWI infarct volume (Tables 2, 3 and Supplemental Tables 6, 7). The GA/HbA1c ratio was also associated with both severe stroke severity (initial NIHSS score > 14) and the highest quartile of DWI infarct volume (Q-100) (Tables 2, 3). We categorized GA and HbA1c into tertiles and analyzed the effect on stroke severity and DWI infarct volume. The highest tertile of GA was associated with both severe stroke symptoms and the highest quartile (Q-100) of DWI infarct volume. The highest tertile of HbA1c was not associated with initial stroke severity but was associated with the highest quartile (Q-100) of DWI infarct volume (Supplemental Tables 10, **11**).

As shown in Table 1, the LAA group was 125, the SVO group was 78 and the CE group was 51 in this study. When the patients were stratified by stroke mechanism, the effects of GA on moderate-to-severe stroke symptoms (NIHSS 6–42) and the highest quartile of DWI infarct volume (Q-100) were statistically significant for only LAA (*p* = 0.04 and 0.01, respectively), whereas they were not significant for SVO or CE in the multivariable analysis (Supplemental Tables 8, **9**).

## Discussion

The main findings of this study were as follows: (1) the higher GA group had severe stroke symptoms and large infarct volume; (2) a higher GA and higher GA/HbA1c ratio could be associated with severe initial stroke severity and higher infarct volume in acute ischemic stroke patients with DM; (3) a high HbA1c, which represents chronic hyperglycemia prior to stroke, was not associated with initial stroke severity but was associated with increasing infarct volume.

Several studies have shown that exposure of cell cultures to rapid glycemic fluctuations produced more oxidative stress and severe cellular damage than exposure to continuous high glucose, thereby aggravating micro- and macroangiopathy (15–18). Several studies on humans also demonstrated that oscillating glucose was more damaging to endothelial function than sustained hyperglycemia in the context of type 2 diabetes (5, 6). Free radicals produced by inadequate intracellular antioxidant processes and oxidative stress seem to account for these phenomena (19). The present study could support the aforementioned ideas. Several studies on stroke outcome have focused on prestroke chronic hyperglycemia (2, 4, 20–25), but the impact of prestroke glycemic variability on acute stroke outcomes has not been investigated. We suggest that our results could increase the interest in the clinical implications of the impact of prestroke glycemic variability on acute stroke outcomes.

In the present study, high GA, reflecting recent glycemic variability (7, 26), could increase the risk of initial severe stroke symptoms and infarct volume. Compared to HbA1c, GA more accurately reflects short-term glucose fluctuation, postprandial hyperglycemia, and fasting hyperglycemia and thus is useful for monitoring glycemic variability (7, 8, 26). Interestingly, we found that among the patients with well-controlled DM (HbA1c <6.5%), approximately half (48.4%) had higher GA levels in the present study. This result indicated that glycemic fluctuations may occur frequently in DM patients who are regarded as having well-controlled glycemia or those with poor drug compliance but without clinical diabetic symptoms. Hence, GA could be clinically more useful for monitoring glycemic control than time-averaged mean glucose concentration represented by HbA1c. Additionally, GA may be recommended in individuals with pathological conditions such as hematologic disease and chronic kidney disease, which account for a large proportion of stroke patients (27–29). Therefore, although GA testing has not been widely used in the laboratory setting, our study carefully suggested that GA could be a useful potential predictor for the acute outcome of diabetic patients with acute ischemic stroke.

The previous results of the associations between HbA1c and initial stroke severity and infarct volumes remain controversial (2, 23). A previous study (*n* = 96) showed that HbA1c could be associated with initial stroke severity and infarct volume in brainstem infarction among DM and non-DM patients. Another previous study (*n* = 375) showed that HbA1c was associated with infarct growth in only non-DM patients with arterial occlusion. Notably, our results showed that HbA1c was not associated with initial stroke severity but was associated with increasing infarct volume in DM patients. However, when we categorized the HbA1c into tertiles, only the highest tertile of HbA1c was associated with the highest DWI infarct volume. We carefully suggest that the small sample size and the heterogeneity of the populations in each study could lead to the disparities in the results. Additionally, the influence of chronic hyperglycemia seems to be different between DM and non-DM patients (21). Further study on this issue with a large population is needed to demonstrate this controversy.

Interestingly, the impact of GA on acute stroke outcomes was different according to the stroke mechanism in this study. Significant associations of GA and moderate-to-severe stroke (NIHSS > 5) and larger infarct volumes were found in only LAA, whereas not in SVO or CE. GA may be an excellent marker of diabetic complications, especially macrovascular disease (30). HbA1c is a good marker of microvascular complications, and it seems to be less useful for macrovascular complications (31). Acute glucose fluctuation has been postulated to contribute to the pathogenesis of atherosclerosis, including cardiovascular complications (5, 6). With regard to this evidence, the particular association between GA and acute stroke outcomes in LAA could be explained. Nonetheless, we should be cautious regarding generalizing the results because of the relatively small sample size. We carefully point out the possibility that GA has different effects on the outcomes according to stroke mechanisms.

Although we evaluated the impact of prestroke glycemic variability measured by GA on acute stroke outcomes for the first time, several limitations of our study should be noted. First, this was a single-center study with a relatively small number of subjects, despite the use of a prospective database. Second, although we controlled for several confounders in our statistical models, we could not completely exclude the effects of unmeasured confounding variables. Third, we measured GA only at baseline and not serially. The detection of serial changes in GA may be more valuable for predicting outcomes of acute ischemic stroke. Fourth, we did not consider conditions of increasing protein metabolism that affect GA levels, such as thyroid dysfunction, liver cirrhosis, nephrotic syndrome, and other specific medical conditions (32). However, age, body mass index, creatinine, serum albumin, and high-sensitivity C-reactive protein levels were not different according to GA levels. Despite data being unavailable for detailed inflammation laboratory tests, thyroid and liver function tests in our database, this lack of differences in laboratory tests could support our results. Recently, alcohol consumption was shown to reduce the level of GA by deteriorating glucose tolerance (33). Unfortunately, alcohol consumption was not available in our registry database. Further study on this issue will include the alcohol consumption in DM patients. Fifth, although GA could be postulated as a marker of recent glycemic variability, validation of GA was not performed in this study. Hence, we should be cautious regarding generalizing our results.

## Conclusions

The present study suggests that prestroke glycemic variability is associated with initial severe stroke symptoms and large infarct volume in DM patients presenting with acute ischemic stroke. Although further studies are warranted to investigate these issues, monitoring prestroke glycemic variability via GA testing during hospitalization could raise the possibility and interest for predicting outcomes in diabetic patients with acute ischemic stroke. Additionally, measured with HbA1c, the clinical significance of GA measurement could be increased.

## Data Availability Statement

The raw data supporting the conclusions of this article will be made available by the authors, without undue reservation, to any qualified researcher.

## Ethics Statement

Collection of clinical information with informed consent in the registry for monitoring and improving the quality and outcomes of stroke care was approved by the local institutional review board (IRB) of Chuncheon Sacred Heart Hospital (IRB no. 2013-03). Use of the registry database and additional review of medical records for this study were approved by the IRB without consent from patients because of the study subject anonymity and minimal risk to patients (IRB no. 2017-89).

## Author Contributions

S-HL: study design, clinical and image data acquisition, analysis and interpretation, and primary responsibility for writing the manuscript. J-HS: study design, clinical and image data acquisition, data interpretation, critical revision of the manuscript for important intellectual content, and supervision of the study. SP, MJ, YK, YJK, and CK: data acquisition and critical revision of the manuscript for intellectual content.

## Conflict of Interest

The authors declare that the research was conducted in the absence of any commercial or financial relationships that could be construed as a potential conflict of interest.

## References

[1] Gonzalez-Moreno EI Camara-Lemarroy CR Gonzalez-Gonzalez JG Gongora-Rivera F. Glycemic variability and acute ischemic stroke: the missing link?. Transl Stroke Res. (2014) 5:638–46. 10.1007/s12975-014-0365-7 25085437

[2] Li H Kang Z Qiu W Hu B Wu AM Dai Y. Hemoglobin A1C is independently associated with severity and prognosis of brainstem infarctions. J Neurol Sci. (2012) 317:87–91. 10.1016/j.jns.2012.02.024 22425018

[3] Hjalmarsson C Manhem K Bokemark L Andersson B. The role of prestroke glycemic control on severity and outcome of acute ischemic stroke. Stroke Res Treat. (2014) 2014:694569. 10.1155/2014/694569 25295219 PMC4175748

[4] Gao Y Jiang L Wang H Yu C Wang W Liu S. Association between elevated hemoglobin A1c levels and the outcomes of patients with small-artery occlusion: a hospital-based study. PLoS ONE. (2016) 11:e0160223. 10.1371/journal.pone.0160223 27486868 PMC4972422

[5] Monnier L Mas E Ginet C Michel F Villon L Cristol JP. Activation of oxidative stress by acute glucose fluctuations compared with sustained chronic hyperglycemia in patients with type 2 diabetes. JAMA. (2006) 295:1681–7. 10.1001/jama.295.14.1681 16609090

[6] Ceriello A Esposito K Piconi L Ihnat MA Thorpe JE Testa R. Oscillating glucose is more deleterious to endothelial function and oxidative stress than mean glucose in normal and type 2 diabetic patients. Diabetes. (2008) 57:1349–54. 10.2337/db08-0063 18299315

[7] Koga M. Glycated albumin; clinical usefulness. Clin Chim Acta. (2014) 433:96–104. 10.1016/j.cca.2014.03.001 24631132

[8] Freitas PAC Ehlert LR Camargo JL. Glycated albumin: a potential biomarker in diabetes. Arch Endocrinol Metab. (2017) 61:296–304. 10.1590/2359-3997000000272 28699985 PMC10118799

[9] Hong S Kang JG Kim CS Lee SJ Lee CB Ihm SH. Fasting plasma glucose concentrations for specified HbA1c goals in Korean populations: data from the Fifth Korea national health and nutrition examination survey (KNHANES V-2, 2011). Diabetol Metab Syndr. (2016) 8:62. 10.1186/s13098-016-0179-8 27579145 PMC5004334

[10] Lindley RI Wardlaw JM Whiteley WN Cohen G Blackwell L Murray GD. Alteplase for acute ischemic stroke: outcomes by clinically important subgroups in the third international stroke trial. Stroke. (2015) 46:746–56. 10.1161/STROKEAHA.114.006573 25613308

[11] Reinholdsson M Palstam A Sunnerhagen KS. Prestroke physical activity could influence acute stroke severity (part of PAPSIGOT). Neurology. (2018) 91:e1461. 10.1212/WNL.0000000000006354 30232251 PMC6202943

[12] Kouzuma T Uemastu Y Usami T Imamura S. Study of glycated amino acid elimination reaction for an improved enzymatic glycated albumin measurement method. Clin Chim Acta. (2004) 346:135–43. 10.1016/j.cccn.2004.02.019 15256314

[13] Inoue K Tsujimoto T Yamamoto-Honda R Goto A Kishimoto M Noto H. A newer conversion equation for the correlation between HbA1c and glycated albumin. Endocr J. (2014) 61:553–60. 10.1507/endocrj.EJ13-0450 24681757

[14] Yazdanpanah S Rabiee M Tahriri M Abdolrahim M Rajab A Jazayeri HE. Evaluation of glycated albumin (GA) and GA/HbA1c ratio for diagnosis of diabetes and glycemic control: a comprehensive review. Crit Rev Clin Lab Sci. (2017) 54:219–32. 10.1080/10408363.2017.1299684 28393586

[15] Risso A Mercuri F Quagliaro L Damante G Ceriello A. Intermittent high glucose enhances apoptosis in human umbilical vein endothelial cells in culture. Am J Physiol Endocrinol Metab. (2001) 281:E924–E930. 10.1152/ajpendo.2001.281.5.E924 11595647

[16] Quagliaro L Piconi L Assaloni R Martinelli L Motz E Ceriello A. Intermittent high glucose enhances apoptosis related to oxidative stress in human umbilical vein endothelial cells: the role of protein kinase C and NAD(P)H-oxidase activation. Diabetes. (2003) 52:2795–804. 10.2337/diabetes.52.11.2795 14578299

[17] Piconi L Quagliaro L Assaloni R Da Ros R Maier A Zuodar G. Constant and intermittent high glucose enhances endothelial cell apoptosis through mitochondrial superoxide overproduction. Diabetes Metab Res Rev. (2006) 22:198–203. 10.1002/dmrr.613 16453381

[18] Del Guerra S Grupillo M Masini M Lupi R Bugliani M Torri S. Gliclazide protects human islet beta-cells from apoptosis induced by intermittent high glucose. Diabetes Metab Res Rev. (2007) 23:234–8. 10.1002/dmrr.680 16952202

[19] Nalysnyk L Hernandez-Medina M Krishnarajah G. Glycaemic variability and complications in patients with diabetes mellitus: evidence from a systematic review of the literature. Diabetes Obes Metab. (2010) 12:288–98. 10.1111/j.1463-1326.2009.01160.x 20380649

[20] Kamouchi M Matsuki T Hata J Kuwashiro T Ago T Sambongi Y. Prestroke glycemic control is associated with the functional outcome in acute ischemic stroke: the Fukuoka stroke registry. Stroke. (2011) 42:2788–94. 10.1161/STROKEAHA.111.617415 21817134

[21] Zsuga J Gesztelyi R Kemeny-Beke A Fekete K Mihalka L Adrienn SM. Different effect of hyperglycemia on stroke outcome in non-diabetic and diabetic patients–a cohort study. Neurol Res. (2012) 34:72–9. 10.1179/1743132811Y.0000000062 22196865

[22] Jia Q Liu G Zheng H Zhao X Wang C Wang Y. Impaired glucose regulation predicted 1-year mortality of Chinese patients with ischemic stroke: data from abnormal glucose regulation in patients with acute stroke across China. Stroke. (2014) 45:1498–500. 10.1161/STROKEAHA.113.002977 24676777

[23] Shimoyama T Kimura K Uemura J Saji N Shibazaki K Elevated glucose level adversely affects infarct volume growth and neurological deterioration in non-diabetic stroke patients, but not diabetic stroke patients. Eur J Neurol. (2014) 21:402–10. 10.1111/ene.12280 24517878

[24] Wu S Wang C Jia Q Liu G Hoff K Wang X. HbA1c is associated with increased all-cause mortality in the first year after acute ischemic stroke. Neurol Res. (2014) 36:444–52. 10.1179/1743132814Y.0000000355 24649851

[25] Lattanzi S Bartolini M Provinciali L Silvestrini M. Glycosylated hemoglobin and functional outcome after acute ischemic stroke. J Stroke Cerebrovasc Dis. (2016) 25:1786–91. 10.1016/j.jstrokecerebrovasdis.2016.03.018 27103269

[26] Suwa T Ohta A Matsui T Koganei R Kato H Kawata T. Relationship between clinical markers of glycemia and glucose excursion evaluated by continuous glucose monitoring (CGM). Endocr J. (2010) 57:135–40. 10.1507/endocrj.K09E-234 19926921

[27] Koga M Kasayama S. Clinical impact of glycated albumin as another glycemic control marker. Endocr J. (2010) 57:751–62. 10.1507/endocrj.K10E-138 20724796

[28] Rondeau P Bourdon E. The glycation of albumin: structural and functional impacts. Biochimie. (2011) 93:645–58. 10.1016/j.biochi.2010.12.003 21167901

[29] Kim KJ Lee BW. The roles of glycated albumin as intermediate glycation index and pathogenic protein. Diabetes Metab J. (2012) 36:98–107. 10.4093/dmj.2012.36.2.98 22540045 PMC3335903

[30] Furusyo N Koga T Ai M Otokozawa S Kohzuma T Ikezaki H. Utility of glycated albumin for the diagnosis of diabetes mellitus in a Japanese population study: results from the Kyushu and okinawa population study (KOPS). Diabetologia. (2011) 54:3028–36. 10.1007/s00125-011-2310-6 21947435

[31] Cohen RM Smith EP. Frequency of HbA1c discordance in estimating blood glucose control. Curr Opin Clin Nutr Metab Care. (2008) 11:512–7. 10.1097/MCO.0b013e32830467bd 18542015

[32] Furusyo N Hayashi J. Glycated albumin and diabetes mellitus. Biochim Biophys Acta. (2013) 1830:5509–14. 10.1016/j.bbagen.2013.05.010 23673238

[33] Inada S Koga M Alcohol consumption reduces HbA1c and glycated albumin concentrations but not 1,5-anhydroglucitol. Ann Clin Biochem. (2017) 54:631–5. 10.1177/0004563216675646 27705886

