# Supplementary material for: Effect of Prestroke Glycemic Variability Estimated Glycated Albumin on Stroke Severity and Infarct Volume in Diabetic Patients Presenting With Acute Ischemic Stroke
Source: Front Endocrinol (Lausanne). 2020 Apr 21;11:230. doi: 10.3389/fendo.2020.00230 (PMC7186307; doi:10.3389/fendo.2020.00230)

Supplemental table 1. Baseline characteristics of study population according to HbA1c levels

|  | Normal HbA1c group  (HbA1c<6.5%)  n=93 | High HbA1c group  (GA≥6.5%)  n=203 | p-value |
| --- | --- | --- | --- |
| Age, years (SD) | 73.4 (10.7) | 69.0 (11.6) | 0.25^†^ |
| Male, (%) | 55 (59.1) | 120 (59.1) | 0.997^*^ |
| BMI, kg/m^2^ (SD) | 23.5 (3.6) | 24.8 (3.6) | 0.83^†^ |
| Interval from onset to visit, hour  median (IQR) | 10.8 (3.1-39.6) | 14.7 (4.8-40.2) | 0.12^‡^ |
| Prior stroke (%) | 33 (35.5) | 49 (24.1) | 0.05^*^ |
| Hypertension (%) | 78 (83.9) | 144 (70.9) | 0.02^*^ |
| Hyperlipidemia (%) | 14 (15.1) | 37 (18.2) | 0.52^*^ |
| Current smoking (%) | 8 (8.6) | 32 (15.8) | 0.10^*^ |
| Atrial fibrillation (%) | 22 (23.7) | 32 (15.8 | 0.11^*^ |
| Prior antithrombotic agents (%) | 55 (59.1) | 82 (40.4) | 0.004^*^ |
| Initial NIHSS score, (IQR) | 5 (2-8) | 4 (2-11) | 0.58^‡^ |
| Stroke mechanism (%) |  |  | ^0.11*^ |
| SVO | 19 (20.4) | 59 (29.1) |  |
| LAA | 36 (38.7) | 89 (43.8) |  |
| CE | 21 (22.6) | 30 (14.8) |  |
| Others | 17 (18.3) | 25 (12.3) |  |
| Reperfusion therapy (%) | 16 (17.2) | 21 (10.3) | 0.13^*^ |
| Ischemic lesions (%) |  |  | 0.60^*^ |
| Supratentorial | 65 (69.9) | 135 (66.5) |  |
| Infratentorial | 28 (30.1) | 68 (33.5) |  |
| Laboratory data |  |  |  |
| Total cholesterol, mg/dL, (SD) | 143.8 (47.7) | 158.5 (41.7) | 0.23^†^ |
| Hemoglobin, g/dL (SD) | 12.8 (2.0) | 13.7 (2.2) | 0.38^†^ |
| Creatinine, mg/dL (SD | 1.2 (0.9) | 1.1 (0.6) | 0.04^†^ |
| Platelet, ×1000/µL (SD) | 218.0 (65.8) | 232.8 (82.2) | 0.28^†^ |
| LDL, mg/dL (SD) | 52.0 (30.2) | 45.3 (32.0) | 0.02^†^ |
| GA≥16%, (%) | 45 (48.4) | 172 (84.7) | <0.001 |
| Initial random glucose, mg/dL (SD) | 147.7 (59.8) | 186.3 (72.6) | 0.06^†^ |
| SBP, mmHg (SD) | 147.7 (26.8) | 149.5 (24.9) | 0.23^†^ |
| Infarct volume, cm^3^, median (IQR) | 0.78 (0.28-14.53) | 1.23 (0.30-20.54) | 0.51^‡^ |

Abbreviations: HbA1c, glycated hemoglobin; SD, standard deviation; IQR, interquartile range; NIHSS, National Institute Health of Stroke Scale; SVO, small vessel occlusion; LAA, large artery atherosclerosis; CE, cardioembolism; LDL, low-density lipoprotein; GA, glycated albumin; SBP, systolic blood pressure

^*^ Calculated using the chi-square test

^†^ Calculated using Student’s t-test

^‡^ Calculated using Mann-Whitney U test

Supplemental table 2 Multinomial logistic regression analysis for high GA (≥16.0%) and initial stroke severity (reference = NIHSS 0-5)

|  | Adjusted OR | 95% CI | P-value |
| --- | --- | --- | --- |
| NIHSS 5-14 |  |  |  |
| Age | 0.999 | 0.91-1.03 | 0.95 |
| Male | 1.09 | 0.59-1.99 | 0.79 |
| Hypertension | 1.07 | 0.30-3.88 | 0.92 |
| Medication for HTN | 0.87 | 0.27-2.79 | 0.81 |
| Prior antithrombotic agents | 0.76 | 0.42-1.39 | 0.38 |
| Statin use | 0.54 | 0.24-1.24 | 0.15 |
| Medication for DM | 0.60 | 0.25-1.41 | 0.24 |
| SVO | ref |  |  |
| LAA | 2.74 | 1.30-2.78 | 0.01 |
| CE | 4.45 | 1.78-11.12 | 0.001 |
| others | 2.54 | 0.94-6.80 | 0.07 |
| Initial random glucose | 0.998 | 0.99-1.003 | 0.40 |
| Serum albumin | 0.52 | 0.24-1.13 | 0.10 |
| HbA1c | 0.97 | 0.75-1.25 | 0.81 |
| GA≥16.0% | 1.25 | 0.62-2.51 | 0.53 |
| NIHSS>14 | | | |
| Age | 1.03 | 0.98-1.08 | 0.22 |
| Male | 0.70 | 0.28-1.76 | 0.45 |
| Hypertension | 8.95 | 1.77-45.39 | 0.01 |
| Medications for HTN | 0.06 | 0.02-0.23 | <0.001 |
| Prior antithrombotic agents | 1.48 | 0.56-3.91 | 0.43 |
| Statin use | 4.29 | 1.39-13.24 | 0.01 |
| Medications for DM | 0.64 | 0.17-2.41 | 0.51 |
| SVO | ref |  |  |
| LAA | 8.06 | 0.92-71.09 | 0.06 |
| CE | 30.65 | 3.12-301.59 | 0.003 |
| others | 29.06 | 2.87-294.61 | 0.004 |
| Initial random glucose | 1.001 | 0.995-1.01 | 0.82 |
| Serum albumin | 0.59 | 0.21-1.68 | 0.32 |
| HbA1c | 1.18 | 0.84-1.65 | 0.34 |
| GA≥16.0% | 7.99 | 1.75-36.45 | 0.01 |

Abbreviations: GA, glycated albumin; NIHSS, National Institutes of Health Stroke Scale; OR, odds ratio; CI, confidence interval; HTN, hypertension; DM, diabetic mellitus; SVO, small vessel occlusion; LAA, large artery atherosclerosis; CE, cardioembolism; HbA1c, glycated hemoglobin

Supplemental table 3. Linear regression analysis showing the correlation between glycated albumin and initial NIHSS scores

|  | B | SE B | β | p | R | R^2^ |
| --- | --- | --- | --- | --- | --- | --- |
| Constant | 10.55 | 4.45 |  |  | 0.40 | 0.16 |
| Age | 0.05 | 0.03 | 0.09 | 0.14 |  |  |
| Male | -0.35 | 0.67 | -0.03 | 0.60 |  |  |
| Hypertension | -0.36 | 0.74 | -0.03 | 0.63 |  |  |
| Stroke mechanism | -1.44 | 0.33 | -0.25 | <0.001 |  |  |
| Initial random glucose | -0.003 | 0.01 | -0.03 | 0.59 |  |  |
| Serum albumin | -1.42 | 0.83 | -0.10 | 0.09 |  |  |
| HbA1c | -0.34 | 0.30 | -0.09 | 0.26 |  |  |
| Glycated albumin | 0.29 | 0.08 | 0.28 | <0.001 |  |  |

Supplemental table 4. Multinomial logistic regression analysis for high GA (≥16.0%) and quartiles of infarct volumes (reference = Q-25)

|  | OR | 95% CI | P-value |
| --- | --- | --- | --- |
| Q-50 |  |  |  |
| Age | 1.04 | 1.01-1.09 | 0.03 |
| Male | 1.72 | 0.79-3.75 | 0.18 |
| Hypertension | 4.11 | 0.60-28.36 | 0.15 |
| Initial NIHSS | 1.18 | 1.004-1.40 | 0.04 |
| SVO | Ref | | |
| LAA | 1.01 | 0.45-2.26 | 0.99 |
| CE | 3.47 | 0.93-12.96 | 0.07 |
| others | 2.33 | 0.64-8.51 | 0.20 |
| Medications for HTN | 0.13 | 0.02-0.83 | 0.03 |
| Prior antithrombotic agents | 1.12 | 0.52-2.42 | 0.77 |
| Statin use | 1.67 | 0.65-4.31 | 0.29 |
| Medications for DM | 1.36 | 0.41-4.44 | 0.62 |
| Initial random glucose | 0.996 | 0.99-1.002 | 0.22 |
| Serum albumin | 1.30 | 0.47-3.61 | 0.62 |
| HbA1c | 1.28 | 0.92-1.78 | 0.15 |
| GA≥16.0% | 1.30 | 0.58-2.91 | 0.53 |
| Q-75 | | | |
| Age | 1.03 | 0.99-1.08 | 0.15 |
| Male | 1.88 | 0.77-4.59 | 0.17 |
| Hypertension | 2.57 | 0.30-21.98 | 0.39 |
| Initial NIHSS | 1.38 | 1.17-1.63 | <0.001 |
| SVO |  | Ref |  |
| LAA | 7.29 | 2.50-21.29 | <0.001 |
| CE | 12.97 | 2.76-60.92 | 0.001 |
| others | 8.99 | 1.93-41.82 | 0.01 |
| Medications for HTN | 0.36 | 0.05-2.62 | 0.31 |
| Prior antithrombotic agents | 0.81 | 0.35-1.89 | 0.63 |
| Statin use | 0.995 | 0.33-3.05 | 0.99 |
| Medications for DM | 1.86 | 0.45-7.66 | 0.39 |
| Initial random glucose | 0.997 | 0.99-1.004 | 0.44 |
| Serum albumin | 1.25 | 0.41-3.81 | 0.70 |
| HbA1c | 1.21 | 0.84-1.73 | 0.31 |
| GA≥16.0% | 1.48 | 0.59-3.69 | 0.41 |
| Q-100 | | | |
| Age | 1.02 | 0.97-1.07 | 0.38 |
| Male | 1.15 | 0.41-3.20 | 0.79 |
| Hypertension | 1.91 | 0.17-21.67 | 0.60 |
| Initial NIHSS | 1.73 | 1.45-2.06 | <0.001 |
| SVO |  | Ref |  |
| LAA | 10.83 | 2.32-50.62 | 0.002 |
| CE | 31.31 | 4.62-211.99 | <0.001 |
| others | 44.13 | 6.63-293.92 | <0.001 |
| Medications for HTN | 0.41 | 0.04-3.90 | 0.44 |
| Prior antithrombotic agents | 0.92 | 0.34-2.45 | 0.86 |
| Statin use | 1.04 | 0.28-3.89 | 0.95 |
| Medications for DM | 1.10 | 0.24-4.91 | 0.91 |
| Initial random glucose | 0.999 | 0.99-1.01 | 0.88 |
| Serum albumin | 0.73 | 0.23-2.36 | 0.60 |
| HbA1c | 1.62 | 1.08-2.41 | 0.02 |
| GA≥16.0% | 3.36 | 1.01-11.21 | 0.048 |

Abbreviations: GA, glycated albumin; NIHSS, National Institutes of Health Stroke Scale; OR, odds ratio; CI, confidence interval; SVO, small vessel occlusion; LAA, large artery atherosclerosis; CE, cardioembolism; HTN, hypertension; DM, diabetic mellitus; HbA1c, glycated hemoglobin

Supplemental table 5. Linear regression analysis showing the correlation between glycated albumin and DWI infarct volume

|  | B | SE B | β | p | R | R^2^ |
| --- | --- | --- | --- | --- | --- | --- |
| Constant | -406.89 | 391.05 |  |  | 0.50 | 0.25 |
| Age | -0.80 | 2.67 | -0.02 | 0.76 |  |  |
| Male | -57.175 | 58.15 | -0.06 | 0.33 |  |  |
| Hypertension | 68.97 | 64.19 | 0.06 | 0.28 |  |  |
| Initial NIHSS scores | 30.17 | 5.13 | 0.33 | <0.001 |  |  |
| Stroke mechanism | 12.85 | 29.22 | 0.02 | 0.66 |  |  |
| Initial random glucose | -0.39 | 0.43 | -0.05 | 0.37 |  |  |
| Serum albumin | -81.89 | 72.43 | -0.06 | 0.26 |  |  |
| HbA1c | 38.76 | 26.28 | 0.11 | 0.14 |  |  |
| Glycated albumin | 22.57 | 7.24 | 0.23 | 0.002 |  |  |

Supplemental table 6 Multinomial logistic regression analysis for high HbA1c (≥6.5%) and initial stroke severity (reference = NIHSS 0-5)

|  | Adjusted OR | 95% CI | P-value |
| --- | --- | --- | --- |
| NIHSS 5-14 |  |  |  |
| Age | 1.01 | 0.98-1.04 | 0.69 |
| Male | 1.22 | 0.64-2.33 | 0.54 |
| Prior stroke | 0.97 | 0.48-1.94 | 0.93 |
| Hypertension | 1.22 | 0.33-4.45 | 0.77 |
| Current smoking | 0.84 | 0.34-2.08 | 0.71 |
| Atrial fibrillation | 0.12 | 0.02-0.85 | 0.03 |
| Prior antithrombotic agents | 0.88 | 0.45-1.72 | 0.71 |
| Medications for HTN | 0.83 | 0.26-2.70 | 0.76 |
| Statin use | 0.55 | 0.24-1.23 | 0.15 |
| Medications for DM | 0.53 | 0.21-1.33 | 0.18 |
| Reperfusion therapy | 3.29 | 1.26-8.63 | 0.02 |
| Interval from onset to arrival time | 1.00 | 0.998-1.001 | 0.74 |
| SVO | ref |  |  |
| LAA | 2.55 | 1.19-5.43 | 0.02 |
| CE | 23.12 | 3.03-176.51 | 0.002 |
| others | 3.58 | 1.32-9.74 | 0.01 |
| Initial random glucose | 0.999 | 0.99-1.004 | 0.56 |
| Creatinine | 0.79 | 0.47-1.34 | 0.39 |
| Low density lipoprotein | 0.994 | 0.99-1.004 | 0.24 |
| HbA1c≥6.5% | 0.88 | 0.44-1.76 | 0.73 |
| NIHSS>14 | | | |
| Age | 1.04 | 0.99-1.09 | 0.11 |
| Male | 0.82 | 0.31-2.17 | 0.69 |
| Prior stroke | 0.997 | 0.35-2.83 | 0.996 |
| Hypertension | 7.59 | 1.47-39.22 | 0.02 |
| Current smoking | 0.72 | 0.12-4.22 | 0.72 |
| Atrial fibrillation | 0.44 | 0.07-2.60 | 0.36 |
| Prior antithrombotic agents | 1.38 | 0.50-3.75 | 0.53 |
| Medications for HTN | 0.047 | 0.01-0.19 | <0.001 |
| Statin use | 5.07 | 1.63-15.79 | 0.01 |
| Medications for DM | 0.96 | 0.24-3.79 | 0.95 |
| Reperfusion therapy | 9.71 | 2.61-36.04 | 0.001 |
| Interval from onset to arrival time | 0.99 | 0.97-1.01 | 0.23 |
| SVO | ref |  |  |
| LAA | 8.60 | 0.64-78.37 | 0.06 |
| CE | 32.52 | 1.88-562.09 | 0.02 |
| others | 39.44 | 3.57-435.13 | 0.003 |
| Initial random glucose | 1.003 | 0.998-1.01 | 0.22 |
| Creatinine | 1.16 | 0.66-2.05 | 0.61 |
| Low density lipoprotein | 1.002 | 0.99-1.02 | 0.76 |
| HbA1c≥6.5% | 2.32 | 0.76-7.14 | 0.14 |

Abbreviations: HbA1c, glycated hemoglobin; NIHSS, National Institutes of Health Stroke Scale; OR, odds ratio; CI, confidence interval; SVO, small vessel occlusion; LAA, large artery atherosclerosis; CE, cardioembolism

Supplemental table 7. Multinomial logistic regression analysis for high HbA1c (≥6.5%) and quartile infarct volumes (reference = Q-25)

|  | Adjusted OR | 95 CI | P-value |
| --- | --- | --- | --- |
| Q-50 |  |  |  |
| Age | 1.04 | 0.998-1.08 | 0.07 |
| Male | 2.12 | 0.93-4.85 | 0.08 |
| Initial NIHSS | 1.15 | 0.98-1.34 | 0.09 |
| Prior stroke | 1.08 | 0.43-2.74 | 0.87 |
| Hypertension | 2.93 | 0.39-21.82 | 0.29 |
| Current smoking | 0.40 | 0.13-1.23 | 0.11 |
| Atrial fibrillation | 10.52 | 0.33-335.90 | 0.18 |
| Prior antithrombotics use | 0.98 | 0.40-2.38 | 0.97 |
| Medications for HTN | 0.17 | 0.03-1.09 | 0.06 |
| Statin use | 1.45 | 0.58-3.59 | 0.42 |
| Medications for DM | 1.62 | 0.45-5.80 | 0.46 |
| Reperfusion therapy | 0.69 | 0.09-5.34 | 0.72 |
| Interval from onset to arrival time | 0.99 | 0.98-0.997 | 0.01 |
| SVO | ref |  |  |
| LAA | 0.90 | 0.39-2.08 | 0.81 |
| CE | 0.31 | 0.01-10.91 | 0.52 |
| others | 1.36 | 0.35-5.21 | 0.65 |
| Initial random glucose | 0.997 | 0.99-1.01 | 0.29 |
| Creatinine | 1.27 | 0.75-2.15 | 0.37 |
| Low density lipoprotein | 0.999 | 0.99-1.01 | 0.93 |
| HbA1c≥6.5% | 2.59 | 1.04-6.48 | 0.04 |
| Q-75 | | | |
| Age | 1.04 | 0.99-1.09 | 0.09 |
| Male | 2.25 | 0.87-5.82 | 0.09 |
| Initial NIHSS | 1.33 | 1.13-1.56 | <0.001 |
| Prior stroke | 0.91 | 0.31-24.90 | 0.36 |
| Hypertension | 2.79 | 0.31-24.90 | 0.36 |
| Current smoking | 1.63 | 0.51-5.23 | 0.41 |
| Atrial fibrillation | 0.72 | 0.02-22.39 | 0.85 |
| Prior antithrombotics use | 0.90 | 0.35-2.31 | 0.83 |
| Medications for HTN | 0.35 | 0.05-2.69 | 0.32 |
| Statin use | 1.03 | 0.35-3.06 | 0.95 |
| Medications for DM | 1.88 | 0.43-8.25 | 0.40 |
| Reperfusion therapy | 2.72 | 0.42-17.66 | 0.29 |
| Interval from onset to arrival time | 1.00 | 0.999-1.001 | 0.82 |
| SVO | ref |  |  |
| LAA | 7.51 | 2.51-22.43 | <0.001 |
| CE | 15.27 | 0.44-525.19 | 0.13 |
| others | 10.53 | 2.24-49.44 | 0.003 |
| Initial random glucose | 0.997 | 0.99-1.004 | 0.39 |
| Creatinine | 0.63 | 0.22-1.77 | 0.38 |
| Low density lipoprotein | 1.00 | 0.99-1.01 | 0.89 |
| HbA1c≥6.5% | 2.77 | 1.01-7.62 | 0.048 |
| Q-100 |  |  |  |
| Age | 1.02 | 0.97-1.07 | 0.43 |
| Male | 1.32 | 0.46-3.78 | 0.60 |
| Initial NIHSS | 1.69 | 1.43-1.99 | <0.001 |
| Prior stroke | 1.26 | 0.40-3.96 | 0.69 |
| Hypertension | 1.90 | 0.17-20.90 | 0.60 |
| Current smoking | 1.15 | 0.28-4.76 | 0.85 |
| Atrial fibrillation | 2.93 | 0.10-85.27 | 0.53 |
| Prior antithrombotics use | 0.77 | 0.27-2.22 | 0.64 |
| Medications for HTN | 0.38 | 0.04-3.46 | 0.39 |
| Statin use | 1.18 | 0.33-4.23 | 0.80 |
| Medications for DM | 1.43 | 0.31-6.73 | 0.65 |
| Reperfusion therapy | 4.80 | 0.72-31.97 | 0.11 |
| Interval from onset to arrival time | 1.00 | 0.999-1.001 | 0.96 |
| SVO | ref |  |  |
| LAA | 9.52 | 2.20-41.16 | 0.003 |
| CE | 7.07 | 0.19-261.73 | 0.29 |
| others | 30.48 | 5.06-183.67 | <0.001 |
| Initial random glucose | 1.003 | 0.996-1.01 | 0.43 |
| Creatinine | 1.39 | 0.78-2.41 | 0.26 |
| Low density lipoprotein | 1.002 | 0.99-1.02 | 0.79 |
| HbA1c≥6.5% | 3.39 | 1.08-10.69 | 0.04 |

Abbreviations: HbA1c, glycated hemoglobin; NIHSS, National Institutes of Health Stroke Scale; OR, odds ratio; CI, confidence interval; SVO, small vessel occlusion; LAA, large artery atherosclerosis; CE, cardioembolism; HTN, hypertension; DM, diabetic mellitus

Supplemental table 8. Multivariable analysis showing the association between GA and initial stroke severity according to stroke mechanisms

|  | LAA | | SVO | | CE | |
| --- | --- | --- | --- | --- | --- | --- |
|  | Adjusted OR^1^ | 95% CI | Adjusted OR^2^ | 95% CI | Adjusted OR^3^ | 95% CI |
| NIHSS 0-5 | reference | | | | | |
| NIHSS >5 | 2.80 | 1.03-7.62 | 0.41 | 0.09-1.88 | 2.42 | 0.43-13.47 |

Abbreviations: GA, glycated albumin; LAA, large artery atherosclerosis; SVO, small vessel occlusion; CE, cardioembolism; OR, odds ratio; CI, confidence interval; NIHSS, National Institutes Health of Stroke Scale

^1^Adjusted for age, sex, hyperlipidemia, serum albumin and glycated hemoglobin which showed p<0.2 from bivariate analyses

^2^Adjusted for age, sex, creatinine, serum albumin and glycated hemoglobin which showed p<0.2 from bivariate analyses

^3^Adujusted for age, sex, total cholesterol, creatinine, low density lipoprotein, systolic blood pressure, initial random glucose, serum albumin and glycated hemoglobin which showed p<0.2 from bivariate analyses

Supplemental table 9. Multivariable analysis showing the association between GA and quartiles of DWI infarct volume according to stroke mechanisms

|  | LAA | | SVO | | CE | |
| --- | --- | --- | --- | --- | --- | --- |
|  | Adjusted OR^1^ | 95% CI | Adjusted OR^2^ | 95% CI | Adjusted OR^3^ | 95% CI |
| Q-25 | reference | | | | | |
| Q-50 | 4.36 | 1.07-17.68 | 0.84 | 0.17-4.26 | 0.52 | 0.04-6.43 |
| Q-75 | 1.65 | 0.48-5.64 | 1.04 | 0.19-5.76 | 4.75 | 0.29-77.44 |
| Q-100 | 10.49 | 1.87-58.81 | 0.38 | 0.06-2.45 | 3.23 | 0.25-41.68 |

Abbreviations: GA, glycated albumin; LAA, large artery atherosclerosis; SVO, small vessel occlusion; CE, cardioembolism; OR, odds ratio; CI, confidence interval

^1^Adjusted for age, sex, hyperlipidemia, serum albumin and glycated hemoglobin which showed p<0.2 from bivariate analyses

^2^Adjusted for age, sex, creatinine, serum albumin and glycated hemoglobin which showed p<0.2 from bivariate analyses

^3^Adujusted for age, sex, total cholesterol, creatinine, low density lipoprotein, systolic blood pressure, initial random glucose, serum albumin and glycated hemoglobin which showed p<0.2 from bivariate analyses

Supplemental table 10. Multinomial logistic regression analysis for tertiles of GA and HbA1c and initial stroke severity (reference = NIHSS 0-5)

|  | Lowest tertile of GA | | Middle tertile of GA | | Highest tertile of GA | |
| --- | --- | --- | --- | --- | --- | --- |
|  | Adjusted OR^1^ | 95% CI | Adjusted OR | 95% CI | Adjusted OR | 95% CI |
| NIHSS 0-5 | reference | | | | | |
| NIHSS 6-14 | - | - | 1.36 | 0.67-2.78 | 1.49 | 0.63-3.57 |
| NIHSS >14 | - | - | 7.13 | 1.48-34.42 | 11.21 | 1.96-64.09 |
|  | Lowest tertile of HbA1c | | Middle tertile of HbA1c | | Highest tertile of HbA1c | |
|  | Adjusted OR^2^ | 95% CI | Adjusted OR | 95% CI | Adjusted OR | 95% CI |
| NIHSS 0-5 | reference | | | | | |
| NIHSS 6-14 | - | - | 0.96 | 0.45-2.02 | 2.30 | 0.62-8.48 |
| NIHSS >14 | - | - | 0.94 | 0.40-2.18 | 3.50 | 0.88-13.92 |

^1^ adjusted for age, male, hypertension, medication for hypertension, medication for DM, statin medication, initial random glucose, albumin, HbA1c, stroke mechanism

^2^ adjusted for age, male, initial stroke severity, prior stroke, hypertension, smoking, atrial fibrillation, medications for hypertension, medications for DM, statin use, pervious antithrombotic agents, reperfusion therapy, time interval from onset to reperfusion, initial random glucose, creatinine, low density lipoprotein and stroke mechanism

Supplemental table 11. Multinomial logistic regression analysis for tertiles of GA and HbA1c and quartiles of DWI infarct volume (reference = Q-25)

|  | Lowest tertile of GA | | Middle tertile of GA | | Highest tertile of GA | |
| --- | --- | --- | --- | --- | --- | --- |
|  | Adjusted OR^1^ | 95% CI | Adjusted OR | 95% CI | Adjusted OR | 95% CI |
| Q-25 | reference | | | | | |
| Q-50 | - | - | 1.86 | 0.75-4.61 | 0.58 | 0.18-1.85 |
| Q-75 | - | - | 2.02 | 0.74-5.56 | 0.55 | 0.15-2.09 |
| Q-100 | - | - | 2.87 | 0.60-13.76 | 4.15 | 1.10-15.62 |
|  | Lowest tertile of HbA1c | | Middle tertile of HbA1c | | Highest tertile of HbA1c | |
|  | Adjusted OR^2^ | 95% CI | Adjusted OR | 95% CI | Adjusted OR | 95% CI |
| Q-25 | reference | | | | | |
| Q-50 | - | - | 1.98 | 0.73-5.39 | 2.64 | 0.82-7.59 |
| Q-75 | - | - | 2.70 | 0.93-7.87 | 1.33 | 0.38-4.64 |
| Q-100 | - | - | 2.06 | 0.58-7.28 | 4.27 | 1.07-17.05 |

^1^ adjusted for age, male, hypertension, medication for hypertension, medication for DM, statin medication, initial random glucose, albumin, HbA1c, stroke mechanism

^2^ adjusted for age, male, initial stroke severity, prior stroke, hypertension, smoking, atrial fibrillation, medications for hypertension, medications for DM, statin use, pervious antithrombotic agents, reperfusion therapy, time interval from onset to reperfusion, initial random glucose, creatinine, low density lipoprotein and stroke mechanism

Supplemental figure 1. Scattered plot showing the correlation between GA and outcomes


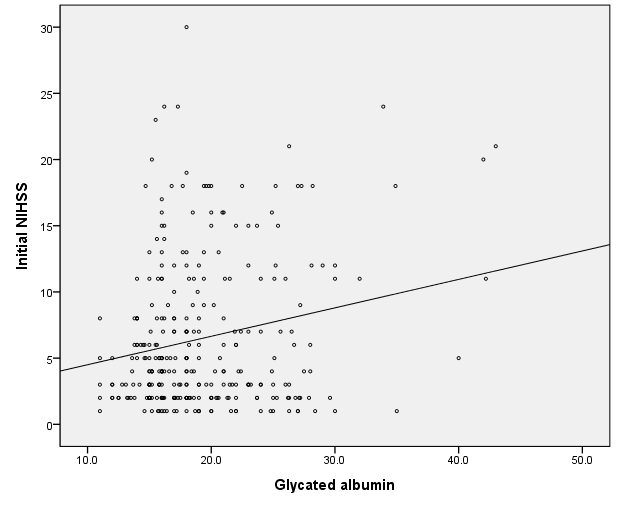

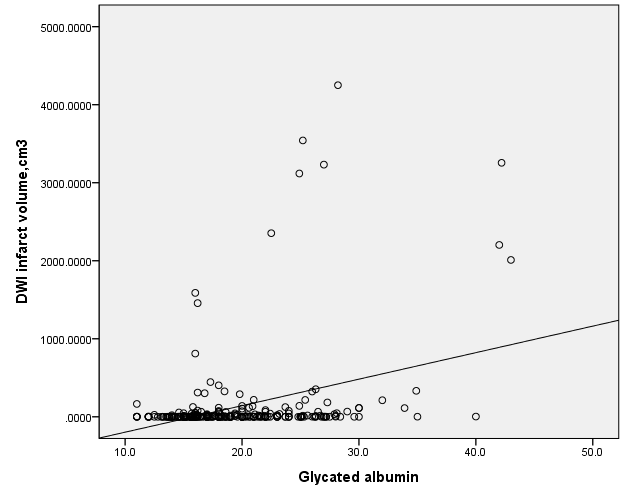

Supplement: Supplementary file 1 [file Data_Sheet_1.docx]
